# Diabetic Wound-Healing Science

**DOI:** 10.3390/medicina57101072

**Published:** 2021-10-08

**Authors:** Jamie L. Burgess, W. Austin Wyant, Beatriz Abdo Abujamra, Robert S. Kirsner, Ivan Jozic

**Affiliations:** Wound Healing and Regenerative Medicine Research Program, Department of Dermatology and Cutaneous Surgery, University of Miami Miller School of Medicine, Miami, FL 33136, USA; jlb452@med.miami.edu (J.L.B.); waw8@med.miami.edu (W.A.W.); Bxa520@med.miami.edu (B.A.A.)

**Keywords:** diabetes, wound healing, diabetic foot ulcer (DFU)

## Abstract

Diabetes mellitus is an increasingly prevalent chronic metabolic disease characterized by prolonged hyperglycemia that leads to long-term health consequences. It is estimated that impaired healing of diabetic wounds affects approximately 25% of all patients with diabetes mellitus, often resulting in lower limb amputation, with subsequent high economic and psychosocial costs. The hyperglycemic environment promotes the formation of biofilms and makes diabetic wounds difficult to treat. In this review, we present updates regarding recent advances in our understanding of the pathophysiology of diabetic wounds focusing on impaired angiogenesis, neuropathy, sub-optimal chronic inflammatory response, barrier disruption, and subsequent polymicrobial infection, followed by current and future treatment strategies designed to tackle the various pathologies associated with diabetic wounds. Given the alarming increase in the prevalence of diabetes, and subsequently diabetic wounds, it is imperative that future treatment strategies target multiple causes of impaired healing in diabetic wounds.

## 1. Introduction

**Figure 1:**
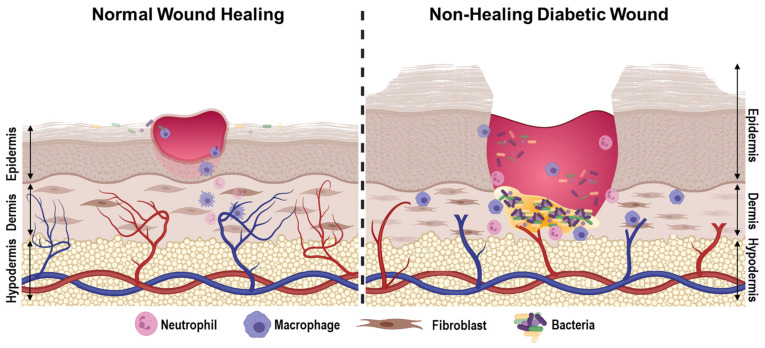
Pathophysiology of diabetic wounds. Diabetic wounds exhibit deregulated angiogenesis, chronically sustained sub-optimal inflammatory response, increased levels of reactive oxygen species, and persistent bacterial colonization that often develops into a hard-to-treat biofilm. Created with BioRender.com, 29 July 2021.

Currently, close to 500 million people are estimated to be suffering from diabetes mellitus (DM), with a predicted startling increase in the upcoming years. In the US alone, over $300 billion is spent annually on both medical costs and as a result of lost workdays due to DM [1,2]. Moreover, one estimate suggests that between one in three to one in every five patients with DM will develop a chronic non-healing wound in their lifetime, such as a diabetic foot ulcer (DFU), with an alarming recurrence rate (40% within one year and 65% within five years) and no reliable methods available to predict its occurrence [3,4]. Considering additional factors identified by Armstrong et al., the overall lifetime incidence of foot ulcers in diabetic patients could be as high as 19–34% [5]. Thus, it is not surprising that a large proportion requires lower limb amputations, affecting patients’ quality of life and requiring costly treatments; it is estimated that the DFU market alone is set to increase from 7.03 billion USD in 2019 to 11.05 billion USD by 2027, making it imperative that more effective diagnostic and treatment strategies are developed to combat this debilitating disease [2,4]. The diabetic foot ulcer has an exceptionally complex pathology due to persistent hyperglycemia and associated diabetic complications, including (1) barrier disruption and infection, (2) high oxidative stress, (3) neuropathy, (4) microvascular complications, and (5) suboptimal chronic inflammatory response, in addition to psychological problems, including a patient’s mental health, self-esteem, and family cohesion (among others) (Figure 1). Below, we will outline recent advances in our understanding of the pathophysiology of diabetic wounds and then review current/upcoming diagnostic and treatment strategies for this devastating disease.

## 2. Pathophysiology Associated with Diabetic Wound Healing

### 2.1. Hyperglycemia

In patients with DM, hyperglycemia can contribute to impaired wound closure and development of DFUs through atherosclerosis, impaired functioning of various skin cells, and peripheral neuropathy. Although hypoglycemia has also been associated with the vascular complications of diabetes [6], most of the literature, and thus this section, focuses on the deleterious effects of hyperglycemia as it relates to the development and progression of DFUs. Hyperglycemia contributes to the development of atherosclerosis, thereby preventing circulating nutrients from reaching wounds, impairing healing [7]. Moreover, in patients with DM, hyperglycemia has been found to be a potential cause of dysfunction of endothelial cells [8], which are critical for the healing of DFUs [9,10] via pressure-induced vasodilation, a response that is normally protective for the skin [8].

In addition to endothelial cells, hyperglycemia also disrupts processes that are critical for re-epithelialization, namely, the protein synthesis, migration, and proliferation of keratinocytes and fibroblasts [11,12,13,14]. In patients with DFUs, the expression of several keratinocyte proteins related to re-epithelization are disrupted, including cytoskeletal keratin proteins (K2, K6, and K10), which are important for keratinocyte differentiation, and a laminin-5 α3 chain precursor protein (LM-3A32), which regulates the binding of epithelial cells to the basement membrane [15]. Subsequently, reduction of LM-3A32 perturbs keratinocyte survival and differentiation and thus re-epithelization [16].

Another mechanism by which hyperglycemia impairs wound healing is via free radical damage as a result of reduced activity of the antioxidant enzymes glutathione peroxidase and superoxide dismutase [17]. This may partly explain why other studies have found that long-standing uncontrolled hyperglycemia is correlated with higher levels of markers associated with the skin aging process, namely, advanced glycation end products (AGEs) and their receptors [11]. Hyperglycemia can also lead to the production of reactive oxygen species (ROS) via the polyol, hexosamine, protein kinase C, and AGE pathways [18]. Although it is understood that ROS are required for the early stages of wound healing [19,20], the imbalance of ROS production has been shown to be detrimental to later stages of wound healing. Specifically, elevated ROS levels can damage the blood supply, metabolism, and structure of peripheral nerves. In affected nerves, this can lead to dysfunction of sensory, motor, and/or autonomic functioning, with each deficit uniquely increasing the risk of developing a DFU [21]. Together, these changes brought on by uncontrolled high blood glucose levels make the skin more susceptible to injury and infection, which impairs wound healing (see the section below on infection).

### 2.2. Neuropathy

In addition to increasing the risk of DFU formation, each type of neuropathy (sensory, motor, and/or autonomic) can uniquely contribute to impaired DFU healing. For example, autonomic neuropathy decreases sweat gland activity, leaving skin dry and cracked, thereby increasing the risk for pruritus and infection, which inhibits wound healing [18]. In addition to dry skin and poor circulation, diabetic neuropathy is, for unclear reasons, associated with pruritus [22,23]. Meanwhile, motor neuropathy increases pressure on the plantar surface of the foot, leading to tissue ischemia and death [18]. Overall, neuropathic skin has a reduced density of neurons and exhibits reduced skin healing [24].

Besides optimizing blood sugar control, patients may prevent dry skin, and thus pruritus, by avoiding exposure to hot water and utilizing moisturizers, particularly ones without perfumes or dyes. Other treatments for improving wound closure in patients with diabetes and neuropathy include antibiotics if an infection is present, as well as debridement and wound cleansing [25]. Recent evidence also suggests that hyperlipidemia, specifically hypertriglyceridemia, may play a role in the development of diabetic neuropathy and therefore, lipid-lowering drugs may prevent or even reverse the damage to nerve fibers in patients with diabetic neuropathy [26]. This approach to prevention is not often employed as therapies targeting neuropathy currently focus on reducing the pressure placed on the foot and relieving the need to itch [25]. Since neuropathy predominantly affects nerves that are dependent on nerve growth factor (NGF) in diabetic patients, exogenous NGF supplementation has demonstrated improved wound contraction, leukocytic chemotaxis, and keratinocyte turnover [27] and, in one study, clinically improved healing [28].

### 2.3. Microvascular Complications

#### 2.3.1. Peripheral Arterial Disease

Peripheral arterial disease (PAD) is prevalent in patients with DFU and contributes to worse outcomes and increased risk of limb amputation [29,30]. One cross-sectional study found PAD in 43% of cases of DFUs [29]. Similarly, a retrospective comparison of patients with Charcot foot found a high prevalence of PAD, which was predicted by the presence of DFUs [31]. There are many revascularization techniques that have shown that reperfusion of the ulcer area in eligible patients decreases amputation risk and death [32,33,34]. One method to evaluate the benefit of revascularization for the treatment of PAD in diabetic patients is with the wound, ischemia, and foot infection (WIFI) classification system developed by the Society for Vascular Surgery. While WIFI Q1–3 cases had approximately 83–87% healing rates, in WIFI Q4 cases, where revascularization had an uncertain benefit, patients had increased limb amputation rates even when revascularization was performed [35]. As another predictor of healing after revascularization, a prospective cohort study found that an increase in >2 °C of surface skin temperature after endovascular therapy is associated with increased wound healing [36].

#### 2.3.2. Hypoxia

As a natural consequence of poor circulation in patients with DM, DFUs result in hypoxic environments. In the setting of hypoxia, the various cell populations of the skin have differential gene expression. Using a cell culture model, Alessandro et al. found endothelial cells and differentiated macrophages encoded genes for angiogenesis, cytokines, and growth factors, while keratinocytes and dermal fibroblasts had gene expression changes for cell metabolism proteins [37]. A recent study monitoring skin hypoxia using flow-mediated skin fluorescence found that lower levels in DFUs corresponded to a worse healing prognosis and other complications [38]. In an attempt to compensate for these hypoxic conditions, hyperbaric oxygen therapy has been extensively used in the treatment of DFUs, though the exact mechanisms of hyperbaric oxygen treatment on DFU gene expression are still under investigation (see below for hyperbaric treatment strategy).

#### 2.3.3. Anemia

In recent studies, anemia has been demonstrated to be prevalent in patients with DM, especially in the setting of DFUs [39,40,41,42,43,44]. However, there are conflicting reports on the correlation between anemia and DFU prognosis. A meta-analysis found that increasing anemia severity was associated with DFU severity and could serve as a predictor of amputation and mortality [45]. Retrospective cohort studies identified anemia as significantly associated with larger, deeper ulcers, more severe infections, high amputation risk, and increased mortality rates [46,47], while observational studies in Nigeria have found anemia to be associated with poor wound healing, amputation, and increased mortality [48,49]. Conversely, other studies found anemia to be a non-significant predictor of clinical outcome for patients with DFUs [50,51,52]; thus, the context under which anemia may be a prognostic factor for DFU wound healing is still debatable and requires further elucidation.

### 2.4. Barrier Disruption and Infection

#### 2.4.1. Transepidermal Water Loss (TEWL)

A healthy skin barrier relies on a well-regulated balance of lipids, cell–cell junctions, antimicrobial peptides, and enzymes to prevent water loss and infection. The uppermost skin layer (stratum corneum) is composed of the terminally differentiated, denucleated keratinocytes filled with keratin fibers and cross-linked envelope proteins called corneocytes that are surrounded by a hydrophobic lipid layer, and as such, protects against transepidermal water loss (TEWL). With age, the skin naturally has decreased lamellar body secretions, depletion of lipids, slower barrier repair, and increased TEWL [53,54]. Though the global stratum corneum water content decreases with age [55], the surface stratum corneum water content has been shown to be similar in both young and aged skin [56].

Diabetic skin has been found to be remarkably similar to aged skin, with decreased lipid content, decreased stratum corneum hydration, and increased AGEs, though some studies noted no significant changes to TEWL [11,57,58] while other studies noted an increase [57,59]. A comparative analysis between humans and rats suggested that in diabetics, a paradoxically insignificant change in TEWL could be due to a decrease in sweating to maintain water loss homeostasis [10]. Similarly, a 2017 case–control study found changes in TEWL in diabetics with a dysfunctional peripheral sympathetic nervous system, while no significant findings were found in cases with sensorimotor neuropathy compared to controls [60]. A recent mouse model study by Horikawa et al. comparing skin dryness in type 1 and type 2 diabetes found that type 1 diabetes increased AGEs and matrix metalloproteinase-9 (MMP-9), leading to a decrease in collagen IV, while type 2 diabetes reduced hyaluronic acid levels and increased inflammatory cytokines levels [61]. Skin hydration appeared to be correlated with microcirculation [62] and was found to be a significant predictor of wound healing when hydration was measured prior to interventions such as recanalization [63]. Additionally, hyperglycemia further increases the risk of infection by changing the distribution of tight junction protein 1, altering epidermis histology, and modifying the basal cell ultrastructure, all of which disrupt the normal function of the skin barrier [64].

#### 2.4.2. Antimicrobial Peptides

In conjunction with maintaining water homeostasis, the structural and functional integrity of the skin is essential for preventing infection. Healthy skin has evolved immune mechanisms, such as antimicrobial peptide (AMP) production, to help regulate the natural skin microbiome and keep pathogens in check. In response to *Staphylococcus aureus* (*S. aureus*) infection in healthy skin, dermal fibroblasts can differentiate into adipocytes to produce cathelicidin (LL-37) [65,66], which has also been demonstrated to promote wound healing by stimulating keratinocyte migration and angiogenesis [67]. However, there is little to no production of cathelicidin in DFUs [68], which contributes to the impaired wound healing phenotype. Expression of other AMPs, such as human β-defensins, have been shown to be upregulated in DFUs, but subsequent AMP production has been proposed to be insufficient for microbial regulation [68,69]. To further complicate matters, AMP production has been shown to be influenced by common drugs used in diabetes treatment, such as in the case of RNase 7 downregulation by metformin [69]. Low AMP production combined with evidence of increased AGEs, impaired lamellar body production, and decreased stratum corneum lipid content in the setting of diabetes sets the stage for an impaired wound healing environment [14]. Recent studies have investigated the potential to utilize AMPs to promote wound healing. Treatment of keratinocytes with 1,25-dihydroxyvitamin D3 induced cathelicidin and human β-defensin 2, increased keratinocyte migration, and showed effective antimicrobial activity [70]. Direct delivery of antimicrobial peptides using hydrogels [71], gold nanoparticles [72], and nanopolymers [73] showed antimicrobial activity and enhanced wound healing using mice and in vitro models.

#### 2.4.3. Bacterial Diversity

With the advent of high-throughput technologies, including 16S rRNA sequencing, microarray, and whole-genome sequencing, the characterization of the diabetic skin and microbiome has expanded. There is growing evidence of microbiome dysbiosis of not only the skin [74] but also the gut [75] of diabetics that may contribute to the development and complications of diabetes. Diabetic skin has been shown to have higher colonization of both *S. aureus* and *S. epidermidis* [74]. A recent analysis of German patients with DFUs identified *Staphylococcus, Pseudomonas,* and *Enterobacteriaceae* as the most common bacterial colonizers [76]. When stratifying DFUs by infection severity, a recent study identified *Staphylococcus* and *Streptococcus* as the most abundant species in mildly to moderately infected DFUs, while more severely infected DFUs had increased bacterial diversity [77]. Bacteria such as *Staphylococcus* and *Streptococcus* express proteolytic factors that disrupt the skin barrier. Specifically, it has recently been shown that SpeB from *Streptococcus* cleaves desmoglein 1 and 3, compromising the epidermal barrier and promoting skin infection [78]. The increased prevalence of *S. aureus* colonization of intact diabetic skin and DFUs contributes to the high rate of diabetic foot ulcers infections [74] and subsequent spread of infection to the bone and bloodstream. In fact, osteomyelitis was identified as a significant predictor of wound healing [48] and amputation [79] in a Nigerian multi-center observational study. Unfortunately, systemic antibiotics have limited delivery to chronic wound sites, especially in the presence of a bacterial biofilm, so new therapies have been focused on topical delivery of drugs with varied release mechanisms [80,81].

#### 2.4.4. pH and Microbiome

While the pH of intact skin has generally not shown a significant difference in diabetics versus controls, one study identified a slightly higher skin pH in the intertriginous areas of diabetics [59,82]. The wound environment of DFUs is significantly more alkaline than acute wounds, contributing to the complex host–microbiome interaction. In a test of different bacterial strains, including *Pseudomonas*, alkaline pH conditions increased biofilm formation [83], with pH also exhibiting differential effects on bacterial resistance to antibiotics [84]. Considering antibiotic resistance testing generally occurs near physiological pH, this difference in bacterial sensitivity should be accounted for when prescribing treatment for an infection found in an abnormally alkaline DFU environment.

### 2.5. Inflammation and Immune System Deficiency in Chronic Wounds

The healing dynamics in acute wounds consist of four overlapping phases, including hemostasis, inflammation, proliferation, and remodeling [85,86]. Diabetic individuals can develop various complications, including chronic wounds such as non-healing DFUs, which arise from perturbation of each stage of wound healing [87]. First, unlike acute wounds, DFUs are characterized by the non-resolving inflammation phase, where a large number of neutrophils and macrophages are found in the wound bed [85,88,89], as well as the chronic release of proinflammatory cytokines including interleukin (IL)-1, IL-6, tumor necrosis factor (TNF)-α, and plasma C reactive protein [85,88,90,91], and bacterial proliferation [88,92] being the most explored factors that contribute to the impaired healing process. Another feature of DFUs is the sustained hypoxia state derived from insufficient angiogenesis, in which its state is strengthened by the continuous inflammatory response, resulting in an increase of ROS and dysfunctional healing process [86,93,94]. The downregulation of connective tissue growth factors in DFUs correlates with decreased levels of transforming growth factor (TGF)-β and collagen levels, delaying wound closure by affecting fibroblast proliferation and vascular cell populations in both mouse models and humans [95,96,97,98]. Studies have shown that overexpression of TNF and downregulation of TGF-β1 in macrophages leads to elevated IL-10 levels, reduced collagen production, and increased tissue damage [87]. Altogether, these factors contribute extensively to the prolonged inflammatory state in DFUs and thus inhibit successful wound closure.

Other studies have shown that the failure to progress from the inflammatory to proliferative could result from the activation of the p38 mitogen-activated protein kinase signaling pathway. In this case, p38 induces the release of cytokines and downregulates miR-21, which is involved in the termination of the inflammatory phase [85,99,100,101]. Recently, studies have elucidated the correlation between angiogenesis and inflammation in the progression of chronic wounds. A possible mechanism involves the downregulation of lncRNA metastasis-associated lung adenocarcinoma transcript 1 (MALAT1) [102] in DFUs, dysregulating angiogenesis by lowering vascular endothelial growth factor (VEGF) expression and increasing inflammation in the wound bed [102,103]. Conversely, induction of MALAT1 via the hypoxia-inducible factor (HIF)-α signaling pathway has been demonstrated to restore regular fibroblast activity and promote wound healing in a diabetic mouse model [102,104].

Furthermore, impaired immune cell function has been well documented in diabetic patients [105] who exhibit impaired phagocytic activity and leukocytes dysfunction [106,107]. Macrophages are widely investigated when studying the immune system in chronic wounds of diabetic humans and mice in part because they produce and release cytokines, are influenced by the surrounding microbiome, and coordinate the transition from the inflammatory to proliferative phase [108,109,110,111]. In acute wounds, as the inflammatory stage is resolved, M1 macrophages are replaced by M2, whereas in DFUs, M1 macrophages continue to predominate the wound microenvironment [108,112,113]. Likewise, in diabetic patients, chronic inflammation causes accumulation of T-cells, which may be the reason for high levels of TNF-α and C-C Motif Chemokine Receptor 4 (CCR4) chemokines, significantly affecting the immune response and facilitating the proliferation of opportunistic pathogens [114,115,116,117]. Studies have also shown that the severity of DFUs may be in part determined by the deficiency of the immune response in DFUs via deregulation of IL-6, macrophage migration inhibitory factor (MIF), and interferon-inducible protein (IP)-10 [118], and a compromised neutrophil response [119,120,121]. In recent years, it has been proposed that high platelet-to-lymphocyte (PLR) and neutrophil-to-lymphocyte ratios (NLR) may be a biomarker for DFU severity [122,123], where high PLR levels reflect the increased platelet activity, inflammation, and the risk for thrombosis and atherogenesis [122,124,125], while high NLR levels lead to the upregulation of cytokines and of proteolytic enzymes that can cause tissue damage [122,123].

### 2.6. Psychological Impacts of Diabetes Mellitus

Although DM can have a negative impact on a patient’s mental health, self-esteem, and family cohesion [126], there are conflicting reports on whether or not a DFU diagnosis impacts a patient’s standard of living [127,128]. Moreover, patients with a DFU do not appear to have significantly worse mental health than those without [127,129]. Nonetheless, there is evidence to suggest that DFUs can lead to emotional distress, reduced quality of life (QoL), and physical dysfunction [130]. Physical dysfunction is one of the most important psychosocial aspects for patients living with a DFU since it may restrict their activities of daily living [131] and prevent them from being able to fulfill their normal family and social roles [132]. Moreover, patients with DFUs have worse QoL than patients who healed without amputation or underwent minor amputation [133], which further solidifies the notion that physical dysfunction is central to the psychosocial experience of patients with DFUs. Even most patients who underwent major transtibial amputation experienced improved quality of life [134]. For patients with DFUs that experience significant negative mental health effects, there are psychological interventions available that may reduce anxiety, depression, and patient global assessment scores [135]. Another intervention, characterized by psychotherapy during hospitalization, reduced anxiety, depression, and scores on the Problem Areas in the Diabetes Scale [136].

## 3. Treatment Strategies

Due to the above-referenced multifactorial pathophysiology of diabetic wounds, DFUs remain a clinical challenge. Wound-healing strategies can fall under standard of care therapies and advanced therapies, with the standard of care treatment involving wound debridement, offloading, and glycemic and infection control, whereas advanced therapies include hyperbaric oxygen therapy (HBOT), wound dressings, negative pressure wound therapy (NPWT), and growth factor therapies including platelet-rich plasma, stem cells, and cell- and tissue-based products [2,4] (Table 1). Considering the clinical need, stimuli-responsive and multifunctional treatment strategies that can accelerate diabetic wound healing are likely to be an important part of future diabetic wound management [1].

### 3.1. Debridement

As part of standard care, debridement of the wound bed helps to reduce bacterial burden, including biofilm, and increase the immune system’s effectiveness, among other mechanisms of action [139]. Whereas the presence of bacterial biofilms in acute wounds act as both a mechanical barrier and as an innate progression of wound healing, uncontrolled biofilm formation can become multidrug-resistant and make it difficult for the healing process to occur in DFUs [139,210,211]. Surgical debridement of wounds is thought to promote healing by removing non-viable tissue and perhaps interact synergistically with other co-administered treatments and is included as the standard of care for DFUs [212,213,214]. In a retrospective study of patients with DFUs treated for biofilm-associated infections, sharp debridement combined with meshed skin grafts and NPWT resulted in a mean wound healing time of 3.5 ± 1.8 weeks [215]. Secondary analysis of debridement modalities found that surgical debridement was associated with shorter healing time [137], though there is a lack of strong evidence for surgical debridement efficacy in promoting wound healing [216]. Other studies in porcine models and in humans have demonstrated that another form of debridement, enzymatic debridement, may decrease wound size, reduce inflammation, and increase granulation tissue, with the caveat that they may require a secondary dressing to penetrate the rooted layers of the wound in order to control the biofilms present [139,140,141]. Likewise, it has been shown that hydro-active dressing soaked with polyhexamethylene biguanide (in humans) can promote macrophage activation in the wound, inhibiting bacterial proliferation and dampening inflammation [138,142,143].

### 3.2. Hyperbaric Oxygen Therapy

Despite lacking a multitude of high-quality trials in the use of HBOT, recent work has emphasized that hyperbaric oxygen therapy can be effective for the treatment of patients with Wagner grade 3 and 4 ulcers [144], showing a concurrent improvement in HbA1c, leukocyte levels, and serum creatinine [145]. While a recent study in rabbits treated with HBOT found no significant changes to the expression of genes involved in wound healing [146], a study using a diabetic mouse model found accelerated wound healing and a significant reduction in MMP-9 levels with treatment [147]. In a small study with 17 patients, hyperbaric oxygen therapy was noted to induce cytoplasmic translocation of HIF-1a and nuclear factor (NF)-kB localization as well as increased VEGF, IL-6, insulin-like growth factor binding protein 3, adiponectin, fibrosis, and angiogenesis while decreasing interferon (IFN)-γ levels [148]. In addition, the prolonged use of hyperbaric oxygen therapy has also been shown to decrease the recruitment and adhesion of neutrophils, increase oxygen dispersion to damaged tissues, reduce inflammation, and accelerate healing in patients with diabetic ulcers [149,150,151]. More recently, studies of topical oxygen therapy rather than HBOT have been shown to promote healing in DFUs and promote an aerobic wound microbiome [152,153].

### 3.3. Negative Pressure Therapy and Off-Loading

Recent randomized controlled trials examining the effectiveness of NPWT in the treatment of DFUs have provided mixed results. Landmark studies suggested when used prior to a wound closure therapy, NPWT in more difficult surgically treated DFUs helped improve overall healing. Recently, however, one study found that there was no significant difference in wound closure between NPWT and standard moist wound care [154], and another study found no significant difference in wound closure between NPWT and a traditional Vacuum-Assisted Closure (VAC(^®^)) Therapy System [155]. NPWT remains a part of standard care. It should be noted, however, that NPWT may provide other benefits beyond wound closure. For example, treatment with NPWT significantly reduced leukocyte count, pain, and systemic inflammatory response; discharge criteria and granulation tissue were also present significantly earlier when using this treatment [156]. Whether NPWT increases or decreases blood flow and oxygenation in treated tissues is controversial, as different investigative techniques have yielded varying results. For example, while using NPWT, laser Doppler showed a significant increase in blood flow [157], thermal imaging revealed no significant change in blood flow [158], and transcutaneous partial oxygen pressure demonstrated a significant reduction in tissue oxygenation levels in DFUs, the effects of which may be beneficial since relative ischemia is a stimulus for neovascularization [159].

Off-loading is the best-studied and most reliable element of standard care for patients with DFUs. The treatment involves reducing foot pressure, specifically high plantar foot pressure, which may help prevent ulcer formation [217]. This is particularly important in patients with neuropathy because walking with elevated plantar pressures has been associated with the presence of ulcers [218]. Casting and non-removable walkers to date have the best results, but recently, “diabetes footwear”, which includes shoes and insoles designed to reduce stress on the foot, has emerged as an option for reducing plantar pressures in patients with DM [160]. A recent systematic review and meta-analysis found that the best footwear for reducing plantar pressures includes the features of metatarsal additions, apertures, and arch profiles [161]. In addition to modifying the footwear of patients with diabetes, there are also surgical versions of off-loading available [162]. Some examples of surgical off-loading include Achilles’ tendon release and foot reconstruction, both of which are designed to optimize the foot for long-term offloading [163]. Some studies have even shown that the healing and amputation rates for patients with DFUs are significantly better with surgical off-loading compared to non-surgical treatment [164].

### 3.4. Growth Factor-Based Therapies

Due to their involvement in basically every phase of wound healing, as well as their general deregulation in chronic wounds, growth factors (including keratinocyte growth factor (KGF)-2, platelet-derived growth factor (PDGF), basic fibroblast growth factor (FGFb), epidermal growth factor (EGF), etc.) have long been considered as potential strategies in diabetic wound healing and have exhibited promise in small animal models of diabetic wound healing [219,220,221]. Likewise, NGF supplementation has demonstrated some potential to promote healing after 5–14 weeks of treatment; however, these results came from a very small sample size [27]. The US Food and Drug Administration has approved only one topical-growth-factor (GF)-based therapeutic, Becaplermin (0.01% Regranex^®^ gel), with efficacy to promote healing of DFUs [165,166,167]. Although growth factor therapies exhibited promising results in vitro and in small animal models in vivo, all but one ultimately failed to achieve efficacy in accelerating diabetic wound closure for a number of reasons. For one, locally prolonged bioavailability and hourly interaction of the ligand with the receptor are necessary for successful wound closure, but wounds are known to be a harsh microenvironment full of proteases and peptidases [222], making it hostile for local GF stability, chemical integrity, and bioavailability [223,224]. This has therefore rendered topical delivery of GF therapy futile without encapsulation into a protective delivery vehicle [225,226]. Moreover, DFUs often exhibit deregulation and mislocalization of GF receptors [227,228,229], as well compartmentalization within microdomains (i.e., caveolae), which prevent activation of downstream signaling events [230,231,232]. Therefore, even if GFs can be delivered to the wound, the lack of available and functional receptors precludes their ability to bind to the appropriate GF receptor and elicit a signaling cascade that will ultimately result in accelerated directional cell migration and subsequent wound closure. Thus, unless these underlying problems are not corrected by future formulations that both encapsulate GFs and allow for their sustained slow release, as well as clear GF receptors from sequestration of specialized membrane microdomains, GF-based therapies will continue to be futile.

### 3.5. Hydrogels/Matrices/Dressings and Skin Substitutes

A standard regimen for treating DFUs is the use of dressings in conjunction with various secondary treatments. In recent years, the use of various types of hydrogels composed of adipose-derived stem cells [168], bone marrow mesenchymal stem cells (MSCs) [169], human adipose stem cells containing hyaluronic acid [170], as well as desferrioxamine-laden silk nanofibers [171] has garnered a lot of attention. Moreover, in a pilot study, Kaufman et al. showed that using a decellularized purified reconstituted bilayer matrix has substantially reduced healing time [172]. Similar results were observed when using the Integra^TM^ Flowable wound matrix [173,174]. Other studies have demonstrated that the combination of extracellular matrix and stromal vascular fraction gels, besides promoting healing, also stimulated collagen synthesis and neoangiogenesis using both mouse models and human subjects [175,176]. In the treatment of neuroischemic DFUs, a multicenter, randomized controlled trial found sucrose octasulfate dressings significantly improved wound closure in 48% of patients compared to the control (30%) [177]. Follow-up studies found sucrose octasulfate dressings improved transcutaneous oxygen pressure, and early treatment of DFUs with these dressings could lower treatment costs while improving wound healing rates [178,179]. A recent review by the Agency for Healthcare Research and Quality has identified 76 skin substitute products currently sold in the United States, with a majority being acellular products made up of decellularized dermal, placental, or animal tissue [233]. The best, highest quality clinical data exist for bioengineered skin substitutes with living cells [233]. Meta-analyses have found that skin substitute treatment of DFUs results in a shorter time to wound closure and lower amputation rate when compared to standard of care [234,235,236].

### 3.6. Platelet Gels and PRPs

For over 30 years, the use of autologous platelet-rich plasma (PRP) and platelet gel products has been reported to accelerate the healing of chronic wounds. This results from the presence of several growth factors, including PDGF, TGF-β1, and EGF, as well as antimicrobial effects that stimulate tissue regeneration, cell proliferation and differentiation, α-degranulation, and chemotaxis [185,186,187,188,189,190]. Interestingly, allogeneic-PRP is much less investigated than autologous-PRP, though it is an effective and safe treatment for diabetic chronic wounds [191,192,193]. Currently, PRP is combined with different activators and used either in injection therapies or gels—for instance, the use of platelet-enriched fibrin in combination with collagen-based dressings [194], thrombin/fibrinogen formulations [195,237], or calcium gluconate [183,184]. The LeucoPatch^®^ device, a PRP activated with fibrin embedded in a leukocyte wound dressing produced by the patient’s own blood, has shown significant improvement in healing outcomes [197,198,199]. Moreover, because it is painless, platelet products have been reported to be more acceptable to patients with DFUs and have stimulated healing more than regular saline dressings that are standard care for non-healing DFUs [196].

### 3.7. Stem Cells

Stem cell therapy for the treatment of DFUs has been a recent topic of great interest. Murine diabetic models have found that the use of adipose- [200], umbilical- [201], bone-marrow- [202], and smooth-muscle [203]-derived stem cells or combination therapies with MSCs [204,205] accelerated wound healing. As diabetic-derived stem cells have an impaired healing phenotype, modification of MSCs by selective gene overexpression such as stromal-cell-derived factor (SDF)-1α [180], overexpression of c-Jun [181], depletion of miR-205-5p [103], or by photobiomodulation [238] have shown promising results in promoting wound healing and provides potential pathways for autologous stem cell treatment. In humans, the injection of autologous micro-fragmented adipose tissue [239] and combination therapies with umbilical cord MSCs improved healing outcomes [240]. A recent meta-analysis found lower amputation rates and increased wound healing in autologous stem cell treatment randomized controlled trials [241]. Cell-free therapies using adipose MSC conditioned media [182] and exosomes [207,208,209] to treat wounds are also in development. Some current clinical trials in progress utilize MSCs derived from the umbilical cord (NCT04104451), human placenta (NCT04464213), and adipose tissue (NCT03865394, NCT03916211). Therapy using allogenic adipose stem cell sheets has shown potential for improving DFU wound healing [168] and is being tested in clinical trials (NCT02619877, NCT03754465, NCT04497805, NCT03370874, NCT04569409). Another clinical trial (NCT00955669) found autologous bone marrow MSCs promoted limb blood flow and healing [206]. With such rapid development in the last couple of years, stem cells could be the next generation of therapies for DFU wound healing.

## 4. Diagnostic Measures

### 4.1. Biomarkers

Biomarker identification is essential for the assessment of DFU healing progress and prognosis. DFU biomarkers can be analyzed on a range of specimens, including tissue biopsies, serum, and wound exudate fluid. Of note, though, specimens must be of sufficient quality for proper biomarker analysis, as one study noted the high prevalence of poor-quality tissue specimens in DFUs, which may affect clinical trial designs [242]. One set of biomarkers of interest are inflammatory biomarkers in DFU osteomyelitis, such as erythrocyte sedimentation rate (ESR) and c-reactive protein (CRP), which was the focus of one recent clinical trial (NCT04025853). However, there is much controversy about the efficacy of inflammatory biomarkers for osteomyelitis. Some studies have shown inflammatory biomarkers correlate with developing osteomyelitis [243] or could be used to monitor response to therapy [244]. Additional studies have found procalcitonin to be a predictor of DFU severity, osteomyelitis occurrence, and amputation risk [245,246,247]. In contrast, other studies noted procalcitonin was not effective at differentiating uninfected and infected DFUs [248], with CRP serving as a more sensitive osteomyelitis biomarker instead [249,250], though ESR and CRP were noted for being unreliable in the setting of sensory neuropathy [251]. Another clinical trial (NCT02927678) utilizing white blood cells—single-photon emission computed tomography/computed tomography found that it could be used for prediction of osteomyelitis remission after 1 year [252]. However, prediction relied on experienced nuclear physicians for analysis, which can significantly differ based on the training level of the physician, a drawback that could be compensated for with the use of a composite scoring system [253].

Other ongoing clinical trials utilize TEWL as a marker for DFU recurrence and tissue biomarkers such as c-myc and phosphorylated glucocorticoid receptor (NCT04591691) for assessing wound healing. While a full evaluation of DFU biomarkers is outside the scope of this review, briefly, some recent reports of wound exudate biomarkers for DFU wound healing include epithelial neutrophil-activating protein (ENA)-78 [254], c-x-c motif chemokine ligand 6 (CXCL6) [255], and MMP-9 [256]. A number of serum biomarkers have been identified, including albumin [257], PLR and NLR [122,258], angiopoietin-like 2 (ANGPTL2) [259], lipoprotein-associated phospholipase A2 and interleukin-18 (IL-18) [260], pentraxin 3 [261], T-cell differentiation markers [262], stem/progenitor cells [263], as well as neutrophil extracellular traps (NETs)-specific markers [264]. Recent genomic analyses of DFU have utilized circRNAs [265,266,267], lncRNAs [268], miRNAs [99,100,269], genetic polymorphisms [270,271,272], cytokine arrays [273], and network maps [274] for the identification of other potential biomarkers for DFU diagnosis and prognosis. With the advent of bioinformatic analyses predicting factors for diabetic complications [275], the integration of computational algorithms with clinical observations and biomarker results will likely become the new standard for DFU care.

### 4.2. Biosensors and Imaging

With technological advancements in chip technology, there has been a rise in the medical application of biosensors to predict clinical outcomes such as DFU prevention and monitoring of wound-healing progress. One clinical trial (NCT02586519) uses a pressure-sensing insole coupled to a smartwatch system to provide real-time feedback on plantar pressure offloading. Reports by the investigators found that patients optimally received one alert every 2 h, and a reduction of DFU recurrence was associated with the use of this smart sensor technology [276,277]. Recent guidelines for DFU prevention have incorporated regular monitoring of foot skin temperature to assess for early signs of inflammation and enable early intervention to prevent ulceration [278]. A number of other wearable devices have been created for thermal foot monitoring, including smart insole systems [279,280] and socks [281,282], though the efficacy of these wearables in DFU prevention or wound-healing monitoring need to be further tested. Interestingly, a thermal foot-monitoring smart mat reported by Frykberg et al. has been shown to predict ulcer development an average of 35 days prior to ulcer presentation [283,284]. In addition to prevention, thermal monitoring has also been shown to be a useful predictor of DFU wound healing. A small observational study by Gethin et al. noted that lowered pH and surface temperature correlated with DFU healing status [285]. In an Australian-based pilot study, authors reported a lower ratio of wound bed area as measured by thermal images is predictive of week 4 DFU healing status [286]. Other sensors are being developed that could perhaps be integrated into dressings to provide feedback on wound temperature and pH [287].

## 5. Conclusions

There has been an incredible increase in knowledge on diabetic wound healing mechanisms in recent years, but there are still unmet needs in clinical diabetic wound management. A better understanding of the changes in wound status would allow diagnoses to be made faster, easier, and cheaper. Personalized management and treatment of diabetic wounds can become possible with smart wound dressings, hydrogels, and other technologies. Close monitoring and timely treatments based on these technologies may be able to prevent non-healing wounds from developing further as patients often fail to realize the severity of their wounds. As the population of diabetic patients increases, there is a growing need for chronic wound management, and further research is needed to uncover how to accelerate diabetic wound healing and improve patients’ quality of life.

## Figures and Tables

**Table 1:** Summary of current treatment strategies for diabetic wounds.

Strategy	Intervention	Origin of Evidence	Reference
Debridement	Surgical debridement	Meta-analysis—human	[137]
	Autolytic debridement	Clinical trial	[138]
Wound Cleansing Solutions	Propylbetaine-polihexanide	Porcine models, clinical trial	[139,140,141]
	Polyhexamethylene biguanide	Clinical trial	[142,143]
Oxygen Therapy	Hyperbaric Oxygen Therapy	Mice, rabbits, clinical trials	[144,145,146,147,148,149,150,151]
	Topical Oxygen Therapy	Clinical trials	[152,153]
Negative Pressure Wound Therapy	Negative Pressure Wound Therapy	Clinical trials, porcine	[154,155,156,157,158,159]
Off-Loading	Diabetic footwear	Meta-analysis—human	[160,161]
	Surgical off-loading	Meta-analysis—human	[162,163,164]
Growth Factor Therapies	Nerve Growth Factor (NGF)	Case report—human	[27]
	Belcaplermin—human-platelet-derived growth factor (PDGF)	FDA approved, Clinical trial	[165,166,167]
Hydrogels	Stem cell hydrogels	Mice, rats, clinical trial	[168,169,170]
	Desferrioxamine-laden silk nanofibers hydrogels	In vitro, rats	[171]
Matrices	Decellularized purified reconstituted bilayer matrix	Clinical trial	[172]
	Acellular dermal matrix	Clinical trial	[173,174]
	Stromal vascular fraction gel	Clinical trial, mice, In vitro	[175,176]
Dressings	Sucrose octasulfate	Clinical trial	[177,178,179]
Skin Substitutes	Apligraf, Dermagraft, etc.	Meta-analysis, clinical trial	[180,181,182]
Platelet Products	Platelet gel	Clinical trial	[183,184]
	Platelet-rich plasma	Clinical trial	[185,186,187,188,189,190,191,192,193,194,195,196]
	Autologous platelet-derived product	Clinical trial	[197,198,199]
Stem Cells	Unmodified stem cells	Clinical trial, mice, rats	[200,201,202,203,204,205,206]
	Modified stem cells	In vitro, mice, rats	[103,180,181]
	Cell-free stem cell therapies	Mice, rats	[182,207,208,209]
	Adipose stem cell sheets	Clinical trial	[168]

## Data Availability

Not applicable.
